# Preparation and activity evaluation of angiotensin-I converting enzyme inhibitory peptides from protein hydrolysate of mulberry leaf

**DOI:** 10.3389/fnut.2022.1064526

**Published:** 2023-02-07

**Authors:** Yu Chen, Yu Zhang, Qianhui Qi, Feng Liang, Nan Wang, Qihe Chen, Xue Li, Suling Sun, Xinquan Wang, Kaiwen Bai, Wei Wang, Yingchun Jiao

**Affiliations:** ^1^College of Agriculture and Animal Husbandry, Qinghai University, Xining, China; ^2^Institute of Agricultural Safety and Nutrition, Zhejiang Academy of Agricultural Sciences, Hangzhou, China; ^3^Key Laboratory of Agricultural Product Information Traceability, Ministry of Agriculture and Rural Affairs of China, Hangzhou, China; ^4^Zhejiang Provincial Key Laboratory of Food Safety, Hangzhou, China; ^5^College of Food Science and Engineering, Bohai University, Jinzhou, China; ^6^Zhejiang Shuren University, Hangzhou, China; ^7^School of Biological Systems Engineering and Food Science, Zhejiang University, Hangzhou, China; ^8^School of Biological and Chemical Engineering, Zhejiang University of Science and Technology, Hangzhou, China

**Keywords:** mulberry leaf protein, hydrolysate, angiotensin-I converting enzyme (ACE), inhibitory peptides, molecular docking

## Abstract

Angiotensin-I converting enzyme (ACE) inhibitory peptides drew wide attention in the food industry because of their natural reliability, non-toxicity, and safety. However, the characteristics of ACE inhibitory peptides obtained from protein hydrolysate of mulberry leaf prepared by Flavourzyme were still unclear. Based on the single-factor test, the Plackett–Burman test and response surface test were used to determine the key factors affecting the ACE inhibition rate in mulberry leaf protein hydrolysate and the optimum conditions of enzymatic hydrolysis. The results showed that the optimum technical parameters were as follows: the ratio of material to liquid is 1: 25 (w / v, g/mL), the Flavourzyme to substrate ratio was 3,000 U/g, the temperature of enzymatic hydrolysis was 50°C, pH was 6.3, and the time of enzymatic hydrolysis was 2.9 h. The ACE inhibitory peptides in the mulberry leaf protein hydrolysates were purified by ultrafiltration and gel filtration, aiming to obtain the highest active component. The 12 peptide sequences were identified by reverse liquid chromatography-mass spectrometry, and then, they were docked to the crystal structure of human angiotensin-I converting enzyme (1O8A), and the interaction mechanisms of 12 peptide sequences and 1O8A were analyzed. The docking results showed that among the 12 peptide sequences, ERFNVE (792.37 Da), TELVLK (351.72 Da), MELVLK (366.72 Da), and FDDKLD (376.67 Da), all had the lowest docking energy, and inhibition constant. The chemosynthetic ERFNVE (IC_50_: 2.65 mg/mL), TELVLK (IC_50_: 0.98 mg/mL), MELVLK (IC_50_:1.90 mg/mL) and FDDKLD (IC_50_:0.70 mg/mL) demonstrated high ACE-inhibitory activity with competitive inhibition mode. These results indicated that the ACE-inhibiting peptides from mulberry leaf protein hydrolyzed (FHMP) had the potential activities to inhibit ACE and could be used as functional food or drugs to inhibit ACE. This work provides positive support for mining the biological activity of mulberry leaves in the treatment of hypertension.

## Introduction

Improving the utilization rate of protein in the food industry has become a main choke point problem (1, 2). Enzymatic hydrolysis of protein to bioactive peptides is a standard method to improve the utilization rate of protein (3). Compared with microbial fermentation, the bioactive peptides obtained by enzymatic hydrolysis were more effective, shorter in reaction time, easy to expand (4), high in economic value, and high in safety (5, 6). In the process of the enzymatic reaction, some short protein sequences that may perform biological functions and physiological activities can be released from proteins (7), and the hydrolysis processing could reduce the interference of starch and other components (8) and achieve a higher conversion rate (9, 10). Thus, it was important to investigate the specific condition of enzymatic hydrolysis to obtain bioactive peptides.

It is of high research value to develop efficient antihypertensive products around botanical angiotensin- I converting enzyme (ACE) inhibitory peptides (11). Hypertension is a standard chronic disease and the most critical risk factor for cardio-cerebrovascular disease (12). The pathogenesis of hypertension is associated with the renin tensin system (Renin-Angiotensin System, RAS) (13, 14). RAS contains two key enzymes: hypertension proteinase (Renin) and ACE (15). Therefore, inhibiting the enzyme activity of Renin and ACE is one of the treatments for hypertension (16, 17). ACE belongs to glycoprotein (18). The substrate of Renin is simple, and angiotensinogen is the only known substrate, so it is difficult to find an inhibitor for renin (19). In recent years, most of the research on antihypertensive peptides mainly focused on ACE. To study ACE inhibitory peptides more thoroughly, the ACE inhibitory peptides after hydrolysis were further separated and purified, and their structure and peptide sequence were identified. Keer Ma et al. (20) prepared ACE inhibitory peptides by hydrolyzing *Moringa oleifera* leaf protein with alkaline protease. After separation and purification, it was found that the peptides with less than 1 kDa had a significant ACE inhibitory effect, and two peptide sequences, Leu - Gly - Phe - Phe and Gly - Leu - Phe - Phe, were obtained by mass spectrometry and amino acid sequence analysis. Zhang et al. (21) digested broccoli with pepsin, and the ACE inhibition rate of 12.48 mg/L crude extract was 55.29%, while that of 0.05 mg/L new peptide Lys - Ser - Val - Leu - Leu - Lys - Phe obtained by centrifugation, ultrafiltration, gel chromatography, and high-performance liquid chromatography was 42.86%. It shows that the active peptides with higher inhibition rates can be obtained by separation and purification.

Mulberry leaf is rich in protein, which accounts for 27% of its dry base weight (22). The concentration of amino acid in mulberry leaf is richer than in ordinary grains and legumes (23). Mulberry leaf protein contains a large number of essential amino acids and non-essential amino acids needed by the human body. It is a new renewable protein resource with rich resources, no cholesterol, and specific edible and medicinal value (24). It has high research and development significance and weight (25). At present, mulberry leaves are mainly used for sericulture. The extraction and utilization of mulberry leaf protein are limited and seasonal. Therefore, it is of great potential to extract functional active peptides from mulberry leaf protein. Due to the development of sericulture, mulberry leaf has a wide planting area and rich resources, so they can be used as raw materials for extracting ACE inhibitory peptides (26). In the present study, mulberry leaf protein was hydrolyzed by Flavourzyme to prepare ACE inhibitory peptides; Plackett–Burman and response surface tests were applied to optimize the hydrolysis conditions of mulberry leaf protein for obtaining the peptides with the highest ACE inhibitory activity. Then, The ACE inhibitory peptides were purified and identified from the hydrolysate of mulberry leaf protein. In addition, the structure-activity relationships of the ACE inhibitor peptides were clarified.

## Materials and methods

### Materials

Mulberry leaves were collected from the Mulberry leaf Garden of Zhejiang Academy of Agricultural Sciences; Coomassie brilliant Blue G-250 was purchased from Shanghai Yuan Ye Biotechnology Co., Ltd.; papain (≥800 U/mg), alkaline protease (≥200 U/mg), acid protease (≥50 U/mg), Flavourzyme (≥20 U/mg) and trypsin (≥250 U/mg) were purchased from Qi Yi Biotechnology (Shanghai) Co., Ltd. ACE, Captopril and *N*-(3-(2-furyl) acryloyl)-L-phenylalanyl-glycyl-glycine (FAPGG) were obtained from Sigma- Aldrich Co (St. Louis, MO, USA). The ACE Kit-WST was purchased from Tong Ren Chemical Co., Ltd. Cytochrome C, aprotinin, bacitracin thymopentin, and Gly - Gly - Gly was purchased from Shanghai Yuan Ye Biochemical Technology Co., Ltd. Acetonitrile was purchased from Sigma (St. Louis, USA) for high-performance liquid chromatography (HPLC).

MELVLK, ERFNVE, FDDKLD, and TELVLK were synthesized by Nanjing Leon Biological technology co., LTD, the purity of which was greater than 95%. Other chemical reagents used were of analytical grade.

### Preparation of mulberry leaf protein

Fresh mulberry leaves were washed and dried in an oven at 40°C for 6 h. and crushed into homogeneous powder by a highspeed pulverizer (ZK-300A, Zhongkehaoyu, Beijing). 100 g mulberry leaves powder was extracted with 1 L 60% ethanol at 40°C for 1 h to remove alcohol-soluble substances. The sediments were obtained by centrifugation at 2,795×*g* for 10 min. The sediments were extracted in 2 L of 0.05 mol/L NaOH solution at 25°C for 2 h by shaking. The protein solution was collected by centrifugation at 2,795×*g* for 10 min and precipitated by adjusting to pH 3.5 using 6 mol/L HCl. The precipitates were recovered by centrifugation and were lyophilized.

### Preparation of protease hydrolysates from mulberry leaf

#### Hydrolysis of mulberry leaf protein using various proteases

Mulberry leaf protein was dissolved in distilled water (10 g/100 mL) and successively hydrolyzed by Papain (pH 6.5, temperature 55°C, enzyme dosage (E/S) 5,000 U/g), Acid protease (pH3.5, temperature 37°C, E/S 5,000 U/g), Alkaline protease (pH8.0, temperature 50°C, E/S 5,000 U/g), Trypsin (pH 8.0, temperature 37°C, E/S 5,000 U/g), and Flavourzyme (pH 7.0, temperature 50°C, E/S 5,000 U/g) under their most suitable reaction conditions for 3 h. Enzymatic reactions were stopped by heating at 95°C for 10 min and the hydrolysate was centrifuged by 11,180×*g* at 4°C. The supernatant was collected and lyophilized to measure the ACE inhibition rate.

#### Determination of molecular weight of protein hydrolysates from mulberry leaf

The molecular weight of mulberry leaf protein and its enzymatic hydrolysate was determined by high-performance liquid chromatography (Waters, USA) equipped with a TSK gel 2000 SWXL (300 mm × 7.8 mm, 5 μm) column. The mobile phase was acetonitrile/water/Trifluoroacetic acid (45/55/0.1, v / v / v), the detection wavelength was 220 nm, the flow rate was 0.5 mL/min, the column temperature was 30°C, and the injection volume was 10 μL. Cytochrome C (12.5 kDa), aprotinin (6.5 kDa), bacitracin (1.45 kDa), thymopentin (679 kDa), and Gly - Gly - Gly (189 kDa) were used as relative molecular weight calibration standards. The log Mw and elution time of the standard sample was used as the standard curve (*y* = –4.5518*x* + 30.551, *R*^2^ = 0.9929).

#### Single factor test

Among the five hydrolysates, the one with the highest ACE inhibition activity was chosen for further optimization. The feed liquid ratio (w/v, 1: 15, 1: 20, 1: 25, 1: 30, and 1:35), enzyme to substrate ratios (E/S, 1,000 U, 2,000 U, 3,000 U, 4,000 and 5,000 U/g), enzymatic hydrolyses times (1.0, 2.0, 3.0, 4.0, and 5.0 h); temperatures (35, 40, 45, 50, 55, and 60°C), and pH (5.0, 6.0, 7.0, 8.0, and 9.0) on the inhibitory activity of ACE were further studied. The supernatant was collected and lyophilized, prepare 5 mg/mL solution, and measure the ACE inhibition rate.

#### Plackett–Burman test

Because Plackett–Burman’s design can quickly and effectively screen out the main influencing factors from many factors, it is widely used in factor-screening tests (27). According to the results of the single-factor test. The effects of temperature (40, 50, and 60°C), time (2, 3, and 4 h), feed liquid ratio (1: 20, 1: 25, and 1: 30 g/mL), pH (6.0, 7.0, and 8.0), and enzyme dosage (2,000, 3,000, and 4,000 U/g) were investigated by Plackett–Burman Test with thirteen independent experiments. Each factor took high (1) and low (–1) levels, took the activity inhibition rate as the response value, selected the PB design of Niss7, and set a blank as the error analysis item.

#### Response surface test

Based on the PB test, The feed liquid ratio (1: 20, 1: 25, and 1: 30 g/mL), pH (6.0, 7.0, and 8.0), and enzymatic hydrolyses time (2, 3, and 4 h), were further optimized by a central composite design with seventeen independent experiments. Three levels for each process parameter are coded as –1, 0, and +1.

### Purification of angiotensin-I converting enzyme peptides from leaf protease hydrolysate

#### Ultrafiltration

Refer to the method of Aondona et al. (28), and make some improvements: A proper amount of hydrolysate was prepared into 0.1 g/mL solution with ultra-pure water, and then separated by ultrafiltration membranes of 5, 3, and 1 kDa. The Fraction was named FHMP-I (<5 kDa), FHMP-II (3–5 kDa), FHMP-III (<3 kDa), FHMP-IV (1–3 kDa), and FHMP-V (<1 kDa) and collected separately. These fractions were then freeze-dried and prepared into 1 mg/mL solution to measure their ACE inhibitory activity.

#### Gel column chromatography

The most active fraction obtained by ultrafiltration was dissolved into ultra-pure water and loaded onto a Sephadex G-10 gel filter column (1.6 cm × 100 cm). The column was then eluted with ultra-pure water at a flow rate of 0.5 mL/min. One tube was collected every 10 min and detected at 280 nm. The elution peaks of each group were collected and lyophilized. Then prepared into 1 mg/mL solution measure the ACE inhibitory activity of the elution peak of each group was determined.

#### Identification of the amino acid sequence

The fractions with the highest ACE inhibitory activity after gel filtration chromatography were desalted on a C18 column, and the amino acid sequences were further identified by Thermo Scientific QE mass spectrometer (Thermo, USA). The peptides were dissolved in 20 μL solution (0.1% formic acid, 5% acetonitrile) and subjected to an LC-MS/MS system with a C_18_ column (2 μm, 100 Å, nano Viper, 75 μm i. d. × 150 mm) coupled to a (PepMap RSLC C_18_) electronic spray ion.

The column flow rate was 500 nL/min. The eluting conditions were as follows: mobile phase A (0.1% formic acid), mobile phase B (0.1% formic acid, 80% acetonitrile (ACN), column temperature of 40°C, linear elution procedure (0 to 40 min, B). Positive ion scanning was used, and the scanning range was 100–2,500 (m/z). Tandem mass spectra were processed by PEAKS Studio version X+ (Bioinformatics Solutions Inc., Waterloo, ON, Canada).

Peptide sequences were identified using the database search by PEAKS Studio X software in combination with manual de novo sequencing to process the MS/MS data and to perform peptide sequencing.

#### Molecular docking analysis

Molecular docking was performed between the identified peptides and the crystal structure of the human angiotensin-I converting enzyme (1O8A) according to the method of Samuchaya et al. (29). The semi-flexible docking of 1O8A to ACE inhibitory peptides was calculated using molecular docking software MOE. The crystal structure of 1O8A (PDB, DOI: 10.2210/pdb1O8A/PDB) was downloaded from the PDB database. The water molecules were removed from 1O8A, and Proton hydrogenation and energy minimization of all molecules was performed to obtain the receptor molecules needed for docking. The peptides obtained by PEAKS Studio X analysis and screening were used as the ligand, and the receptor pocket of 1O8A was used as the docking target for molecular docking. The peptide with a better docking effect was screened from the obtained results to analyze the interaction between mulberry leaf protein ACE inhibitory peptides and 1O8A.

#### Peptide synthesis

Based on the molecular screening of peptide sequences, selected peptides were synthesized by China Peptides Co., Ltd. (Shanghai, China) for further analysis.

#### Determination of angiotensin-I converting enzyme inhibitory kinetics

According to the previous method of Shufang Ye (30) to explore the inhibition type of ACEI peptide of mulberry leaf protein, with several modifications. In brief, different concentrations of substrate FAPGG (0.00, 0.20, 0.40, 0.80, 1.6 mM) were reacted with different concentrations of ACE inhibitory peptides for a period of time, without inhibitors as control. The different concentrations of ACE inhibitory peptides were prepared according to their IC_50_ value and half of IC_50_. Lineweaver–Burke plots were constructed using GraphPad Prism version 8 software (GraphPad Software, San Diego, CA, USA).

#### Determination of angiotensin-I converting enzyme inhibition rate of mulberry leaf protein and its enzymatic hydrolysate

The ACE inhibitory activity was determined by ACE Kit-WST (Institute of Tongren Chemistry, Japan). Taking ultra-pure water as blank control, the inhibitory activity of ACE was calculated.


$$P_{\mathrm{inhibitory}\,\mathrm{rate}\%}=\frac{A_{\mathrm{Black1}}-A_{\text{Sample}}}{A_{\mathrm{Black1}}-A_{\mathrm{Black2}}}\times 100\%$$

### Statistical analysis

Each experimental procedure was performed three times, and data were expressed as the mean ± standard deviation. Statistical analyses were conducted by one-way analysis of variance using IBM SPSS 20 (SPSS Inc., Chicago, USA). Duncan’s test was applied to determine the significance of multiple groups. The *P*-value <0.05 was considered statistically significant.

## Results

### Angiotensin-I converting enzyme inhibitory activity and molecular weight of protein hydrolysates from different proteases

**FIGURE 1:**
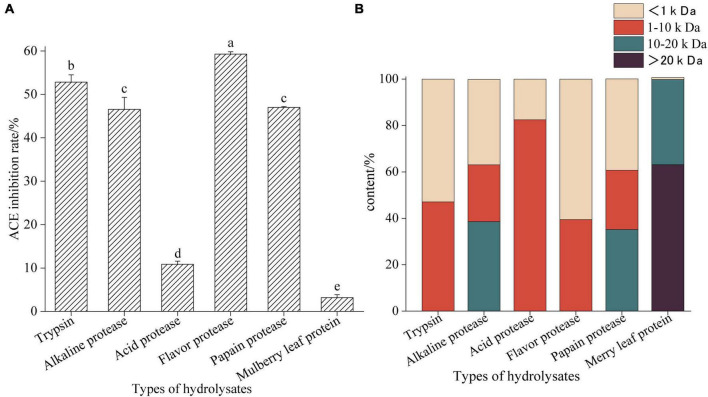
Angiotensin-I converting enzyme inhibition activities of mulberry leaf protein hydrolysates hydrolyzed by different proteases **(A)** and molecular weight distribution **(B)**. Means denoted by a different letter indicate significant differences between treatments (*P* < 0.05).

Figure 1 shows the ACE inhibitory activity and molecular weight distribution of mulberry leaf protein hydrolyzed by five proteases. Compared with other hydrolysates, the enzymatic hydrolysate of Flavourzyme showed the highest ACE inhibitory activity (*P* < 0.05), and its molecular weight was mostly <1 kDa. The molecular weight of mulberry leaf protein is mostly above 10 kDa, but the ACE inhibitory activity is very low.

The content of peptides with molecular weight less than 1 kDa with Trypsin hydrolysate is high, and its ACE inhibitory activity is 52.84 ± 1.25 (*P* < 0.05), which indicates that Trypsin treatment will destroy the protein structure of mulberry leaf protein. Therefore, the results show that different enzymes have different abilities to degrade mulberry leaf protein (31). In addition, we found that the ACE inhibitory activity of the hydrolysate was higher when there were more components with molecular weights less than 1 kDa. This result was similar to that of the chickpea peptide studied by Xuemei Ma (32), indicating that the lower molecular weight with the higher activity of the peptide.

### Selection of technological parameters for protease hydrolysis of mulberry leaf protein

#### Effect of temperature on the inhibition rate of protein peptides angiotensin-I converting enzyme from mulberry leaf

**FIGURE 2:**
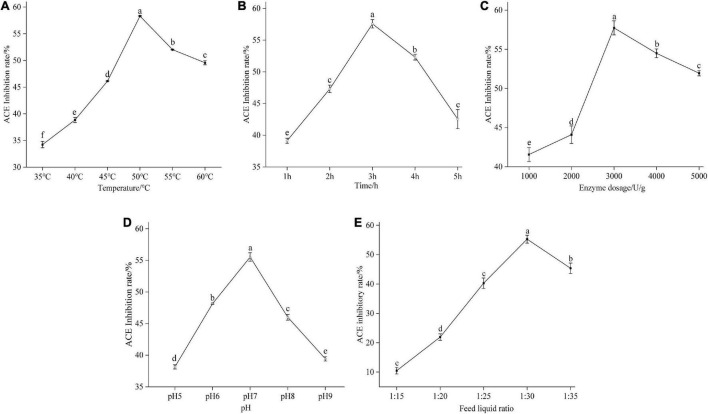
Effect of temperature **(A)**, time **(B)**, enzyme dosage **(C)**, pH **(D)**, and feed liquid ratio **(E)** on the degree of the ACE inhibitory rate of mulberry leaf protein hydrolysate. Means denoted by a different letter indicate significant differences between treatments (*P* < 0.05).

The effect of temperature on the enzymatic reaction was significant (33). When the temperature increases, the reaction speed is accelerated; on the other hand, the increased temperature causes the protease to lose its activity which reduces the reaction rate. In general, the optimum temperature of enzymatic reaction is the result of the equilibrium of these two processes. In this experiment, the temperature gradient was 35, 40, 45, 50, 55, and 60°C. Under these conditions, mulberry leaf protein was hydrolyzed for 3 h, as shown in Figure 2A. With the increase of hydrolysis temperature, the degree of protein hydrolysis and the inhibition rate of ACE in mulberry leaf increased at first and then decreased, indicating that the activity of Flavourzyme increased with the increase of temperature. When the temperature reached 50°C, the inhibition rate of ACE reached the highest, 58.31%. When the temperature reached 55 and 60°C, the inhibition rate of ACE activity decreased gradually, which was 52.0 and 49.6%, respectively. The difference was significant (*P* < 0.05). Therefore, the optimum temperature of enzymatic hydrolysis is 50°C.

#### Effect of enzymatic hydrolysis time on angiotensin-I converting enzyme inhibition rate of mulberry leaf protein peptides

As shown in Figure 2B, the enzymatic hydrolysis time had a significant effect on the inhibition rate of the ACE activity of mulberry leaf protein hydrolysates. With the extension of enzymatic hydrolysis time, the ACE inhibition rate of mulberry leaf protein increased at first and then decreased. The inhibition rate of ACE was only 39.1% at 1.0 h and 47.3% at 2.0 h (*P* < 0.05). However, when the enzymatic hydrolysis time reached 3.0 h, the inhibition rate of ACE continued to increase to 57.6%, which was the maximum. The inhibition rate of enzymatic hydrolysis for 4.0 and 5.0 h began to decrease, and the difference was significant (*P* < 0.05). It may be that the time of enzymatic hydrolysis is too long, which leads to the excessive enzymatic hydrolysis of mulberry leaf protein and a decrease in activity. Therefore, the appropriate time of enzymatic hydrolysis was selected as 3.0 h.

#### Effect of enzyme addition on the inhibition rate of protein peptides angiotensin-I converting enzyme from mulberry leaf

It can be seen from Figure 2C that enzyme dosage had a significant effect on the inhibition rate of ACE of mulberry leaf protein hydrolysates (*P* < 0.05). When E/S was 1,000 U/g, the ACE inhibition rate was 41.5%. With the increase of E/S, the ACE inhibition rate of mulberry leaf protein hydrolysates increased gradually, and the changing trend was larger. The inhibition rate of ACE in the enzyme hydrolysates reached 57.7% when E/S was 3,000 U/g. The analysis of significant differences showed that the ACE inhibition rate of mulberry leaf protein hydrolysates with E/S of 3,000 U/g was significantly higher than that of 4,000 and 5,000 U/g. Therefore, when the E/S is 3,000 U/g, enzyme dosage and feed liquid ratio are just in the saturated state of supply and demand balance (34). Therefore, in the following experiment, enzyme dosage was selected as 3,000 U/g.

#### Effect of pH on the inhibition rate of protein peptides angiotensin-I converting enzyme from mulberry leaf

The optimum pH is an important parameter of enzymatic reaction (35). When pH is 5.0, 6.0, 7.0, 8.0, and 9.0, the inhibition rate of ACE is shown in Figure 2D. After hydrolysis for 3.0 h, with the increase of pH, the ACE inhibition rate of protease hydrolysate increased at first and then decreased. When the pH of protease hydrolysate was 5.0, the ACE inhibition rate of protease hydrolysate was only 38.1%. When pH approached the optimal pH 7.0 of the enzyme, the catalytic efficiency of the enzyme increased, and the ACE inhibition rate of the hydrolysate reached a peak of 55.5%, and then the ACE inhibition rate of the hydrolysate began to decrease. There was a significant difference in the inhibition rate between pH 8.0 and pH 7.0 (*P* < 0.05). When pH increased to 9.0, the inhibition rate of ACE decreased significantly to 39.4%. Therefore, when the enzymatic hydrolysis of pH exceeded the optimum pH range of Flavourzyme, the catalytic activity of the enzyme decreased, which was the same as the change of ACE inhibitory activity of rice protein hydrolysates studied by Wei Kang et al. (36) with pH.

#### Effect of feed liquid ratio on the inhibition rate of mulberry leaf protein peptides angiotensin-I converting enzyme

Figure 2E showed that the feed liquid ratio has a significant effect (*P* < 0.05) on the ACE inhibitory activity of mulberry leaf protein hydrolysates, and the ACE inhibition rate of mulberry leaf protein hydrolysates increases significantly and then decreases with the increase of feed liquid ratio. When the feed liquid ratio was 1: 15, the ACE inhibition rate of mulberry leaf protein hydrolysates was 27.1 ± 2.37%; when the feed liquid ratio was 1: 20, the ACE inhibition rate significantly increased to 39.72 ± 0.75% (*P* < 0.05); when the feed liquid ratio reached 1: 25, the ACE inhibition rate of mulberry leaf protein hydrolysates reached the maximum value of 50.3 ± 1.45% (*P* < 0.05). However, with the continuous decrease of the feed liquid ratio, the ACE inhibition rate of mulberry leaf protein hydrolysates began to decrease. Therefore, the feed liquid ratio were selected as 1: 25 in the following study.

#### Screening and analysis of key factors in the Plackett–Burman test

Based on single factor test, taking the ACE inhibition rate of mulberry leaf protein hydrolysates as the main evaluation index, the Plackett–Burman factor screening test design and main effect analysis were carried out on temperature, time, feed liquid ratio, pH, and enzyme dosage. The results are shown in Tables 1, 2.

**TABLE 1:** Plackett–Burman design and corresponding results.

Test number	A Temperature (°C)	B Time (h)	C Feed liquid ratio (g/mL)	D pH	E Enzyme dosage (U/g)	ACE inhibitory rate (%)
1	40	4	1:30	8	2,000	50.09
2	40	2	1:30	6	4,000	53.13
3	40	2	1:20	8	2,000	62.42
4	40	4	1:30	6	4,000	46.71
5	40	2	1:20	6	2,000	60.67
6	40	4	1:20	8	4,000	55.66
7	60	4	1:30	6	2,000	50.77
8	60	2	1:30	8	4,000	59.84
9	60	2	1:30	8	2,000	61.72
10	60	2	1:20	6	4,000	56.27
11	60	4	1:20	6	2,000	52.76
12	60	4	1:20	8	4,000	53.52

A, B, C, D and E respectively represent the five factors that affect the ACE inhibition rate in Plackett–Burman Test: temperature, time, ratio of material to liquid, pH, and enzyme dosage.

**TABLE 2:** Coefficient estimates of variables in PB design.

Factor	Coefficient estimates	Standard error	*F*-value	*P*-value
A Temperature (°C)	0.5167	0.5386	0.9202	0.3745
B Time (h)	–3.71	0.5386	47.49	0.0005
C Feed liquid ratio (g/mL)	–1.59	0.5386	8.68	0.0257
D pH	1.91	0.5386	12.60	0.0121
E Enzyme dosage (U/g)	–1.11	0.5386	4.23	0.0853
			*R*^2^ = 0.924	*R*_*ad*_^2^ = 0.8624

Table 2 showed that the factors affecting the ACE inhibition rate of mulberry leaf protein hydrolysates were B > D > C > E > A. That is, time > pH > feed liquid ratio > Enzyme dosage > temperature. The effect of time on the ACE inhibition rate of mulberry leaf protein hydrolysates was extremely significant (*P* < 0.01), while the effects of pH and feed liquid ratio on the response value were significant (*P* < 0.05). Therefore, time, pH, and feed liquid ratio were selected for the further response surface design. For the non-significant factors such as E and A, the optimal level of single factor test was selected in the follow-up experiment, that is, enzyme dosage 3,000 U/g and the temperature was 50°C.

#### Response surface test results

Based on single-factor results and the Plackett–Burman factor screening test, time, pH, and feed liquid ratio were selected as independent variables, ACE inhibition rate as the main response value, 3 factors, and 3 horizontal Response surface Test was designed. The results are shown in Table 3, and the significant analysis of variance is shown in Table 4.

**TABLE 3:** Design and results of Box–Behnken experiments.

				ACE inhibitory rate (%)	
Run	A Feed liquid ratio (g/mL)	B pH	C Time (h)	Measured value	Predicted value
1	1:20	7	4	58.07	57.71
2	1:20	7	2	57.84	57.7
3	1:20	6	3	56.76	56.98
4	1:20	8	3	56.86	57.13
5	1:25	8	4	56.51	56.59
6	1:25	7	3	63.03	62.93
7	1:25	7	3	62.04	62.93
8	1:25	8	2	59.17	59.03
9	1:25	7	3	63.13	62.93
10	1:25	6	2	58.72	58.63
11	1:25	7	3	63.16	62.93
12	1:25	6	4	56.76	56.89
13	1:25	7	3	63.28	62.93
14	1:30	7	2	58.47	58.83
15	1:30	6	3	56.38	56.11
16	1:30	8	3	56.28	56.06
17	1:30	7	4	54.5	54.64

A, B, and C respectively represent three factors that have influence on ACE inhibition rate in RSM: Feed liquid ratio, pH and time.

**TABLE 4:** Analysis of variance of the fitted regression model.

Source	Sum of squares	Degree of freedom	Mean square	*F*-value	*P*-value
Model	134.24	9	14.92	64.88	<0.0001
A (Feed liquid ratio, g/mL)	1.90	1	1.90	8.27	0.0238
B (pH)	0.0050	1	0.0050	0.0217	0.8869
C (time, h)	8.74	1	8.74	38.00	0.0005
AB	0.0100	1	0.0100	0.0435	0.8407
AC	4.41	1	4.41	19.18	0.0032
BC	0.1225	1	0.1225	0.5328	0.4891
A^2^	50.52	1	50.52	219.76	<0.0001
B^2^	35.26	1	35.26	153.39	<0.0001
C^2^	21.20	1	21.20	92.22	<0.0001
Residual	1.61	7	0.2299		
Lack of fit	0.5919	3	0.1973	0.7756	0.5655
Pure error	1.02	4	0.2544		
Cor total	135.84	16			
				*R*^2^ = 0.9982	*R*^2^_*adj*_ = 0.9729

In the table, AB, AC, BC, A^2^, B^2^, and C^2^ respectively indicate the regression relationship between the ACE inhibition rate of the dependent variable and several analytical test indexes (dependent variables) in RSM.

Based on the regression analysis of Table 3 data by Design-ExpertV8.0.6 software, the quadratic polynomial regression equation with ACE inhibition rate (Y) as response value was obtained as follows:


$$\text{Y}=62.93-0.4875\,A+0.0250\,\text{B}-1.05\,C-0.0500\,\text{AB}\,-\,1.05\,\mathrm{AC}-0.1750\,\text{BC}-3.46\,A^{2}-2.89\,B^{2}-2.24\,C^{2}$$

The statistical analysis for the model (Table 4) showed that the *P* < 0.0001, indicating that the model has reached a very significant level, the “R-Squared” was 0.9982 and the “adj R-Squared” was 0.9729, which indicates that there is a high correlation between the test value and the fitting value, and. the model can explain at 97.3% of the variation in the data. The “lack of fit” was not significant (*P* > 0.05) indicating that the equation of the model fits the data well and can better reflect the relationship between the respective variables and the response value. Therefore, the regression equation can be used to fit and predict the test results. The differences in the interactive term AC were extremely significant, according to the *P* values of the factors in Table 4. Which indicates that the interaction between A and C was significant.

#### Response surface intuitive diagram analysis of the interaction among factors

**FIGURE 3:**
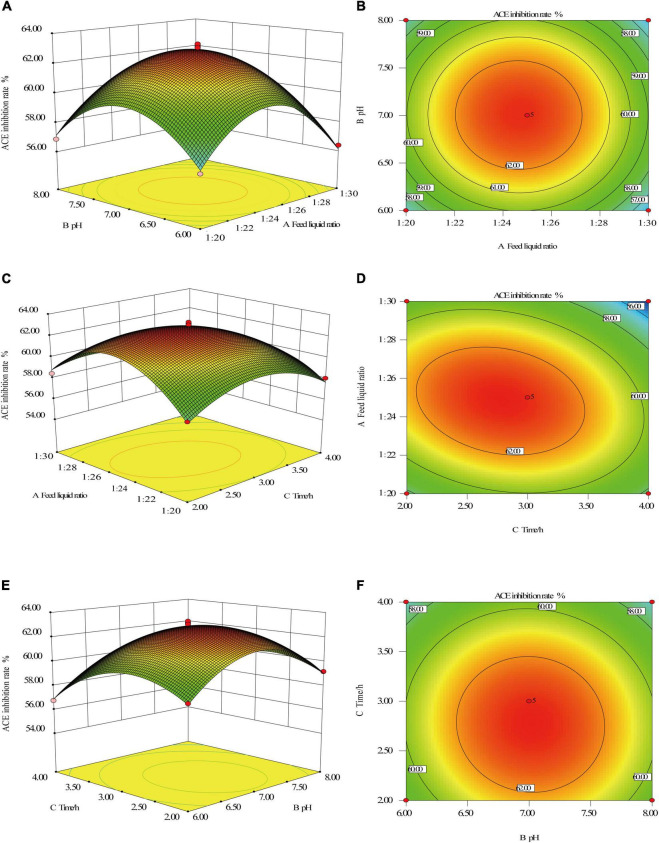
Response surface intuitive diagram of the interaction between factors. Feed liquid ratio and pH **(A,B)**, Feed liquid ratio and enzymolysis time **(C,D)**, and pH and enzymolysis time **(E,F)**.

Based on the results of the analysis of variance, the interaction of substrate and liquid ratio, pH and time was analyzed by Design-ExpertV8.0.6 software, and the results are shown in Figure 3. The response surface diagram can directly show the influence of factor interaction on the response value, while the shape of the contour can reflect the strength of the interaction, the circle indicates that the interaction is not significant, and the oval indicates that the interaction between the two is significant (37, 38).

It can be seen from Figure 3 that the contours of the feed liquid ratio and pH, pH, and enzymatic hydrolysis time are approximately circular, indicating that the interaction between feed liquid ratio and pH, pH, and enzymatic hydrolysis time is not significant. To obtain the maximal ACE inhibitory activity of the hydrolysates. The optimal conditions were extracted by Design-Expert V8.0.6 software, and the optimum conditions were feed liquid ratio at 1: 25, enzyme dosage at 3,000 U/g, enzymatic hydrolysis temperature at 50°C, pH at 6.3, enzymatic hydrolysis time at 2.9 h. Under this enzymatic hydrolysis process, the predicted value of the ACE inhibition rate is 60.6%.

#### Verification of the best process conditions

Under the above optimal process conditions, three verification tests were carried out, and the results showed that the inhibition rate of ACE was 60.3, 60.9, and 60.3%, and the average clearance rate was 60.5%, which was close to the theoretical value of the model. It shows that the model can be used to predict the preparation process of ACE inhibitory peptides from mulberry leaf protein hydrolysates.

#### Ultrafiltration isolation and activity determination of mulberry leaf protein angiotensin-I converting enzyme inhibitory peptides

**FIGURE 4:**
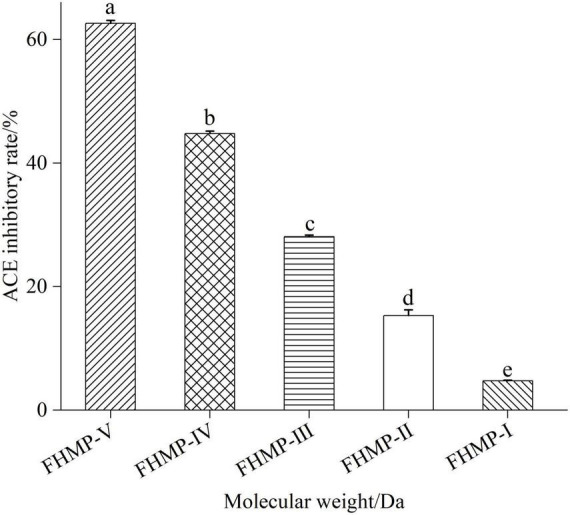
Results of ACE inhibitory activity of peptides after ultrafiltration. Means denoted by a different letter indicate significant differences between treatments (*P* < 0.05). FHMP-I (<5 kDa), FHMP-II (3–5 kDa), FHMP-III (<3 kDa), FHMP-IV (1–3 kDa), FHMP-V (<1 kDa).

As shown in Figure 4, the mixture of proteolytic peptides from mulberry leaf was separated by ultrafiltration to obtain six components, namely, FHMP-I (<5 kDa), FHMP-II (3–5 kDa), FHMP-III (<3 kDa), FHMP-IV (1–3 kDa), FHMP-V (<1 kDa). The results showed that the smaller the molecular weight of the peptide, the higher the ACE inhibitory activity.

At the concentration of 1.0 mg/mL, the ACE inhibition rate of FHMP-V was 62.63 ± 0.48% and higher than those of the other five components. Previous studies have shown that some plant protein peptides can inhibit ACE activity, such as sesame peptide samples (39) and Moringa peptides (20). Compared with these peptides, mulberry leaf peptides showed excellent inhibitory activity on ACE, especially when the molecular weight was less than 1 kDa. Therefore, FHMP-V was selected for further separation and purification.

#### Purification and activity results of mulberry leaf protein angiotensin-I converting enzyme inhibitory peptides gel Sephadex G-10

**FIGURE 5:**
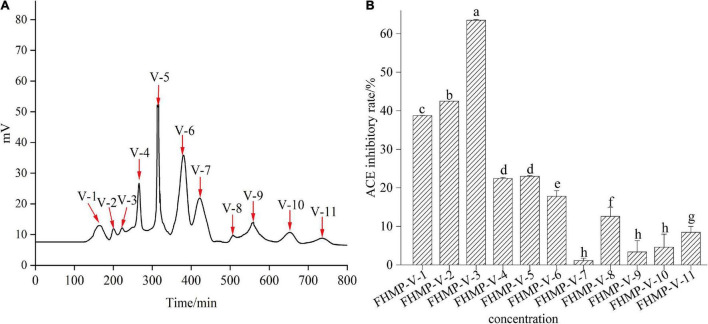
The curve of FHMP-V gel chromatography **(A)** and the ACE inhibitory activity of 11 mixed components (V-1 to V-11) **(B)**. Means denoted by a different letter indicate significant differences between treatments (*P* < 0.05).

Gel filtration chromatography is usually used to separate and enrich peptides (40). According to the difference in molecular weight, Sephadex G-10 gel filtration column chromatography was used to separate FHMP-V ACE inhibitory peptides. Figure 5A shows that FHMP-V is divided into 11 components: FHMP-V-1, FHMP-V-2, FHMP-V-3, FHMP-V-4, FHMP-V-5, FHMP-V-6, FHMP-V-7, FHMP- V-8, FHMP-V-9, FHMP-V-10, FHMP-V-11. Each component was collected and freeze-dried to investigate its ACE inhibitory activity. As shown in Figure 5B, component FHMP-V-3 showed the strongest ACE inhibitory activity. At the test concentration of 0.5 mg/mL, the ACE inhibition rate of component FHMP-V-3 was 63.5 ± 0.21%. Which was higher than that of FHMP-V (62.6 ± 0.48%; 1.0 mg/mL). Most reported peptides with ACE inhibitory activity have low molecular weights, and the sequence lengths are generally less than 12 residues (41). Wu et al. (42) found out that molecular weights of peptides with the strongest ACE inhibitory rate were below 1 kDa. In the Sephadex G-10 gel chromatography, the component FHMP-V-1 contained more peptides with larger molecular weights and weaker activity. The final eluted component FHMP-V-3 with the smallest molecular weights showed the highest ACE inhibition rate and was selected for further identification.

#### Identification of peptides in mulberry leaf protein hydrolyzed-V-3 fractions by RPLC-MS/MS combined with *de novo* sequencing

Since the ACE inhibitory activity of FHMP-V-3 was higher than that of other fractions, it was inferred that it might contain more active ACE inhibitory peptides. Peptides were identified using LC-MS/MS analysis and de novo sequencing on Peak Studio 8.0. Twelve peptides were simultaneously identified in the FHMP-V-3 fraction by LC-MS/MS combined with de Novo sequencing, all sequences are listed in Table 5.

**TABLE 5:** Peptide sequences with fractions higher than 80 in FHMP-V-3 were identified by LC-MS/MS combined with *de novo* sequencing.

Sequence	Mass (m/z)	RT (min)	Length	Score
MELVLK	731.4251	18.17	6	97
YDPVVR	747.3915	25.45	6	96
ERFNVE	792.3766	27.17	6	91
FDDKLD	751.3388	31.57	6	89
TELVLK	701.4323	30.52	6	89
EALERL	729.402	20.7	6	88
LLLALLL	767.5521	22.64	7	84
LLLLLLL	809.599	14.31	7	84
VLRLSL	699.4643	13.57	6	84
WLDGVL	701.3748	15.85	6	82
EPPRYP	757.3759	16.98	6	80
LTLLLR	727.4956	10.71	6	80

Sequence, mass (m/z), RT (min), length, and score indicates the peptide sequence, molecular weight, the number of amino acids in the peptide segment and the confidence score of the peptide segment with a score higher than 80 in FHMP-V-3 identified by LC-MS/MS combined with de novo sequencing respectively.

#### Molecular docking of the peptide sequence to the 1O8A binding site

To further explore the interaction between polypeptide sequences and ACE, 12 polypeptide sequences were used for molecular docking with the crystal structure of the human angiotensin-converting enzyme (1O8A).

Low molecular weight angiotensin-converting enzyme inhibitory peptides have been shown to have better antihypertensive activity (43, 44) and exert their antihypertensive effect by binding to the active site of angiotensin-converting enzyme (45). Molecular docking between 1O8A and 12 peptide sequences was performed by MOE software. In this method, binding energy can be used to indicate the binding between the ligand and the receptor.

**FIGURE 6:**
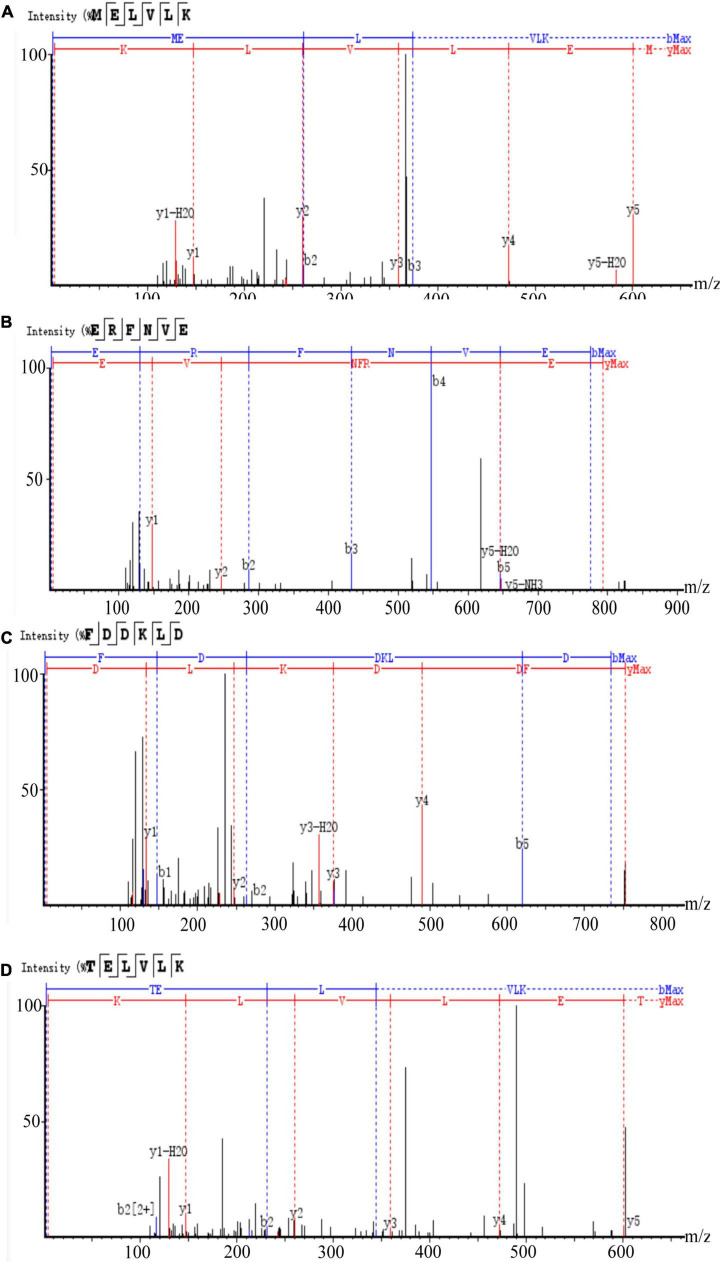
LC-MS chromatogram of FHMP-V-3: **(A)** MELVLK; MS/MS spectrum at m/z 366.72, **(B)** ERFNVE; MS/MS spectrum at m/z 397.19, **(C)** FDDKLD; MS/MS spectrum at m/z 376.67, and **(D)** TELVLK; MS/MS spectrum at m/z351.72. All MS/MS spectra were performed under positive mode.

**FIGURE 7:**
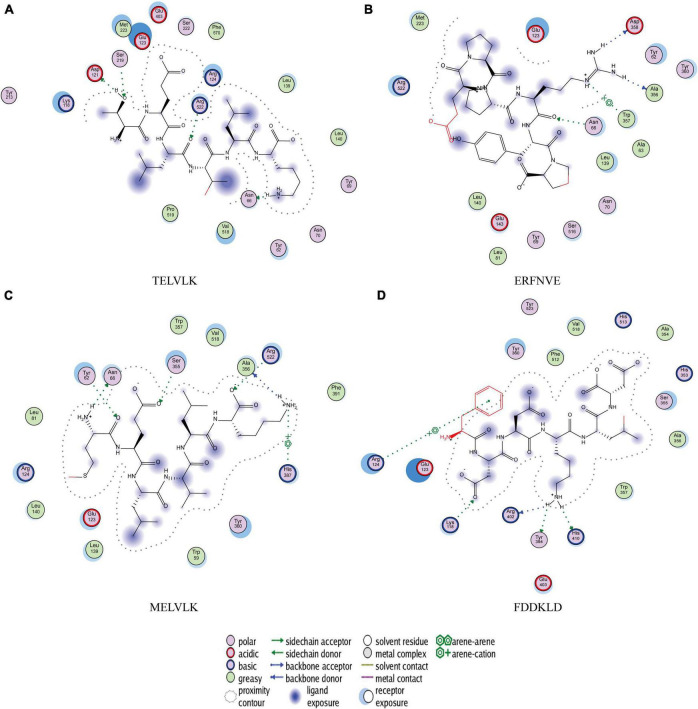
Molecular docking results of **(A)** TELVLK; **(B)** ERFNVE; **(C)** MELVLK; **(D)** FDDKLD, 2D structures of the peptides illustrating the poses for the binding of the peptides to the molecular surface of ACE.

The results of molecular docking show that the ACE inhibition rate of these peptides is realized by forming hydrogen bonds with the residues inside and outside the active center, closing the active center, or distorting the catalytic configuration. Among the 12 peptide sequences, there are eight peptides with binding energy less than –16 with residues around the active site of 1O8A, as shown in Table 6. As the activity of the peptide is also related to its binding active site, the peptide sequences with more than three binding bonds to active sites were further selected, namely ERFNVE, TELVLK, MELVLK, and FDDKLD (Figure 6). It can be seen from Figure 7 that these four peptide sequences are deeply bound into the binding cavity of ACE.

**TABLE 6:** Eight peptides and their binding sites.

Sequence	E-score	Site	Molecular structural formula
EPPRYP	–18.55	Tyr360, Arg402	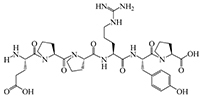
EALERL	–17.28	Tyr62, Glu384, Arg522	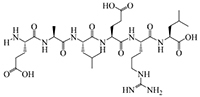
TELVLK	–17.19	Asp121, Ser219, Arg522, Asn66	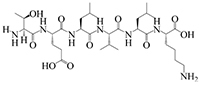
KLLEAAK	–16.96	Tyr62, Arg522	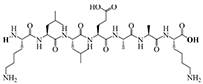
ERFNVE	–16.91	Trp59, Lys118, Tyr394, Arg522, Tyr523	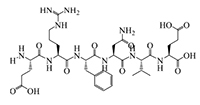
LRVVSE	–16.91	Asn66, Trp357	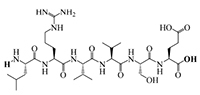
MELVLK	–16.7	Asn66, Tyr62, Ser355, Arg522, His387	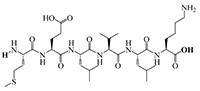
FDDKLD	–16.6	Arg124, Lys118, Tyr394, His410, Arg402	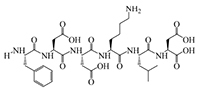

Sequence, e-score, site and molecular structural formula respectively represent the peptide sequences, binding energies, binding sites and molecular structural formulas of eight peptides with binding energies greater than –16.

TELVLK had four hydrogen bonding forces at Asp121, Ser219, Arg522, and Asn66 (Figure 7A); ERFNVE interacts through four hydrogen bonds with residues Trp59, Arg522, Lys118, and Tyr523 of 1O8A (Figure 7B); MELVLK forms five hydrogen bonds at Asn66, Tyr62, Ser155, Arg522, and His387 in 1O8A (Figure 7C); FDDKLD forms five hydrogen bonds at Arg124, Lys118, Tyr394, Arg402, and His410 in 1O8A (Figure 7D). Through molecular docking, we can quickly find the target sequence that can inhibit ACE, so that we can directly synthesize the peptide sequence and explore its mechanism.

#### Kinetics of enzyme inhibition

**FIGURE 8:**
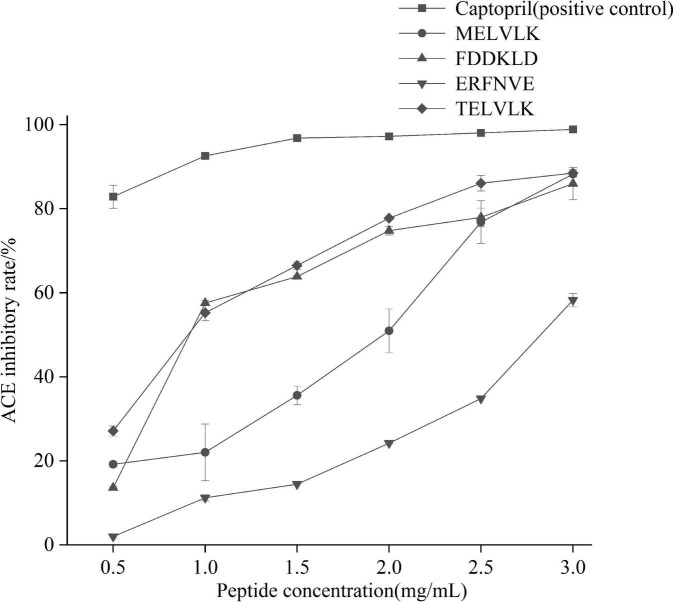
Angiotensin-I converting enzyme inhibitory activity of synthetic peptides.

All the peptides were commercially synthesized and their ACE activity was determined (Figure 8). It confirmed that these peptides had an ACE activity with different IC_50_ values (Table 7).

**TABLE 7:** Kinetics parameters of ACE-catalyzed reactions in four peptides.

Reaction kinetic parameters	ERFNVE (mg/mL)		MELVLK (mg/mL)		FDDKLD (mg/mL)		TELVLK (mg/mL)	
	2.65	1.32	1.89	0.940	0.700	0.350	0.980	0.490
Km (Mm) or K′m (mM)	0.268	0.132	0.480	0.450	0.241	0.248	0.270	0.260
Vmax or V′max (ΔA340 nm)	0.0710	0.0710	0.165	0.236	0.216	0.112	0.0950	0.189
Ki (mM)	0.646		0.829		0.153		0.241	

Km (Mm) or K′m (mM) represent the Mie constant; Vmax or V′max (ΔA340 nm) represent the reaction rate when the enzyme is saturated with the substrate; Ki (mM) represent the inhibition constant, respectively.

**FIGURE 9:**
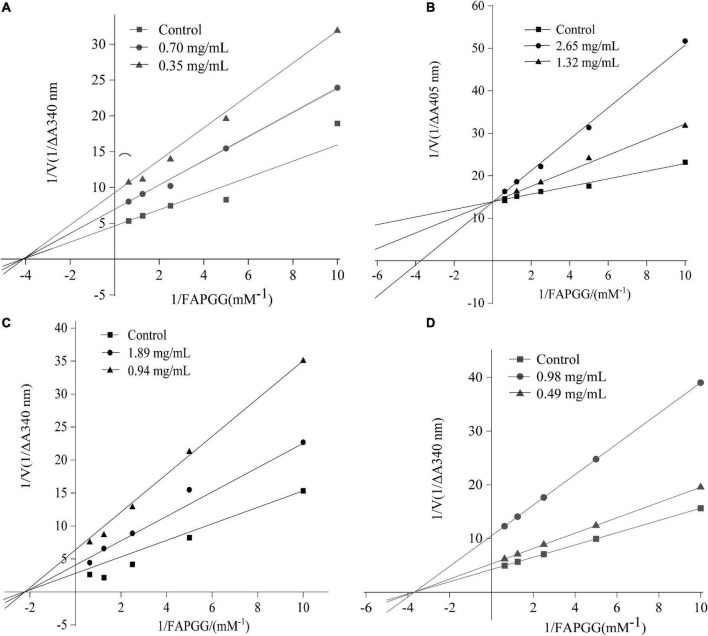
Lineweaver–Burk plot of ACE inhibition by the peptides. **(A)** FDDKLD; **(B)** ERFNVE; **(C)** MELVLK; and **(D)** TELVLK.1/[S] and 1/[V] represent the reciprocal substrate concentration and velocity, respectively.

To determine the inhibitory kinetic mechanism of the ACE inhibitory peptides obtained from mulberry leaf protein, Lineweaver–Burk plots were generated, and the results are illustrated in Figure 9. The parameters of Km (Michaelis constant), Vmax (maximum velocity), and Ki (inhibitor constant) were obtained from the graphs (Table 7). IC_50_ values were also calculated for each peptide, and the values ranged from 0.70 to 2.65 mg/mL.

As shown in Figure 9B, the intersection of the lines at the 1/V axis indicated that the peptides ERFNVE is competitive inhibitors of ACE. On the other hand, Figures 9A,C,D showed that the peptides FDDKLD, MELVLK, and TELVLK have lines of the intersection at the 1/[s] axis, suggesting their non-competitive inhibition kinetics. In the non-competitive inhibition, Vmax decreases as peptide concentration increases, but Km remains unchanged. The Ki values of the peptides were observed to range between 0.153 mg/mL for FDDKLD and 0.829 mg/mL for MELVLK. The inhibitor constant Ki indicates the potency of the inhibitor to the enzyme to form the enzyme-inhibitor complex and the inhibitor does not affect the binding of the enzyme to the substrate, but only reduces the concentration of the effective enzyme. Peptides with low Ki values have a higher potential to inhibit ACE.

## Discussion

In the present study, HMP was hydrolyzed by five proteases. The ACE inhibitory activities of the five hydrolysates were different at the concentration of 1mg/mL, we inferred that this might be related to the restriction sites of different proteases. The biological activities of peptides are generally inextricably related to the N-terminal and C-terminal residues in the polypeptide chain (40). Studies have shown that the key factor affecting ACE inhibition by peptides is hydrophobic amino acids, and hydrophobic amino acids account for a large proportion of ACE-inhibitory peptides (46). The main peptide bond of Flavourzyme is related to the carboxyl group of aromatic amino acids (47); the target peptide bond of Alkaline protease is its carboxyl-terminal hydrophobic amino acid (48). Therefore, we speculate that Flavourzyme can destroy the protein structure of mulberry leaf protein and completely degrade mulberry leaf protein so that the enzymolysis products are rich in oligopeptides and small molecular polypeptides of less than 1 kDa. However, these peptides contain a lot of hydrophobic amino acids, which may be the reason for the high activity of Flavourzyme hydrolysates. In addition, the molecular weight (MW) of the peptide plays an important role in ACE inhibitory activity (49). We found that the smaller the molecular weight of the hydrolysate, the higher the ACE inhibitory activity. The results are similar to the kenaf (*Hibiscus cannabinus L.*) seed studied by Nurul Dhania et al. (50). Therefore, we used Flavourzyme to hydrolyze HMP. The high inhibitory activity of mulberry leaf protein ACE inhibitory peptide was obtained under optimal conditions.

Ultrafiltration is commonly used to purify peptides from protein hydrolysates (51). The peptides from the HMP hydrolysate were purified by membrane filtration followed by gel filtration. Component FHMP-V-3 showed the strongest ACE inhibitory activity. At the test concentration of 0.5 mg/mL, the ACE inhibition rate of component FHMP-V-3 was 63.5 ± 0.21%. Which was higher than that of FHMP-V (62.6 ± 0.48%; 1.0 mg/mL). Peptides were identified using LC-MS/MS analysis and de novo sequencing on Peak Studio 8.0. Four peptides (ERFNVE, TELVLK, MELVLK, and FDDKLD) were simultaneously identified in the FHMP-V-3 fraction by LC-MS/MS combined with de Novo sequencing. Similar analysis has ever been used in many other reports for the identification of bioactive peptides (52–54). According to the report, the presence of amino acid residues G, I, L, and V at the N-terminus or P, Y, R / K, and W at the C-terminus of the ACE-inhibitory peptides is more conducive to the inhibition of ACE. The N-and-C-termini of the 4 peptides identified in this study were similar to those previously reported for ACE-inhibitory peptides (55–57). The properties of MELVLK and TELVLK are also consistent with the view proposed by Fujita et al., that the positive charge on the C-terminal basic residue or basic residue (R/K) contributes greatly to the inhibitory potency (58, 59). In the peptide sequences we studied, the content of hydrophobic amino acids is very high. For example, ERFNVE contains two hydrophobic amino acids, Phe and Val, accounting for 33%; TELVLK contains two hydrophobic amino acids, Val and Leu, accounting for 50%; MELVLK contains three hydrophobic amino acids, Met, Val, and Leu, accounting for 66%, and FDDKLD contains two hydrophobic amino acids, Phe and Leu, accounting for 33%.

To clarify the relationship between the inhibitory peptide and the action of ACE. Molecular docking of the peptide sequence with 1O8A was performed. The amino acid binding sites in the cavity were composed of 133 amino acid residues, including three active pockets, S1 (Ala354, Glu384, and Tyr523), S2 (Gln281, His353, His513, Lys511, and Tyr520), and S1′ (Glu162) and Zn^2 +^, as reported in the literature (60). The results of molecular docking showed that the ACE inhibition rate of these peptides was achieved by forming hydrogen bonds with residues inside and outside the active center, closing the active center, or twisting the catalytic configuration, four peptide sequences interact with ACE through hydrogen bonds. We found that TELVLK could form 4 hydrogen bonds with four amino acid residues of ACE including Asp121, Ser219, Arg522, and Asn66 (Figure 7A); ERFNVE could form 4 hydrogen bonds with four amino acid residues of ACE including Trp59, Arg522, Lys118 and Tyr523 of 1O8A (Figure 7B); MELVLK could form 5 hydrogen bonds with five amino acid residues of ACE including Asn66, Tyr62, Ser155, Arg522, and His387 in 1O8A (Figure 7C); FDDKLD could form 5 hydrogen bonds with five amino acid residues of ACE including Arg124, Lys118, Tyr394, Arg402, and His410 in 1O8A (Figure 7D). More important, the residues Tyr523 belong to the ACE active site S1 (61), which indicated that ERFNVE could exhibit high ACE-inhibitory activity by interacting with the key active site S1 of ACE, corresponding to its competitive inhibition modalities (Figure 9). In the competitive inhibition, the Vmax remains the same for the two peptide concentrations including control, but the Km increases since a higher concentration of substrate is needed to compete with the competitive inhibitors to reach Vmax. The unchanged Km reflects that the inhibitor does not affect the binding of the enzyme to its substrate, but just lowers the concentration of usable enzymes. This result is the same as the molecular docking result. Therefore, the results of molecular docking can also be used as a predictive means for identifying active peptides as well as molecular verification. Through molecular docking, we can quickly find the target sequence that can inhibit ACE, so that we can directly synthesize the peptide sequence and explore its mechanism.

Furthermore, the ACE inhibitory activities of peptides within MW 500–800 Da are higher than that of peptides above 800 Da (62). In the present study, the molecular weights of four peptides were in the range of 350–800 Da, suggesting that these peptides may have higher ACE inhibitory activity. We have chemically synthesized the amino acid sequences of four peptides and confirmed their activities. Certainly, the synthetic peptides exhibited different ACE inhibition activities owing to their different amino acid sequences. Among them, MELVLK exhibited the highest ACE inhibition activity. Thus, our results might contribute toward incorporating ACE inhibitory peptides in functional foods or drug formulations for the therapy of diseases associated with hypertension. However, it still needs more key technologies to make the preparation process more scientific and efficient. Further study their ACE inhibitory activity and activity mechanism in vivo, to better service for the utilization of mulberry leaf protein hydrolysates.

## Conclusion

In this study, the preparation process of ACE inhibitory peptides by Flavourzyme was optimized by a single factor experiment, Plackett–Burman experiment, and response surface experiment, and 12 peptide sequences were obtained from FHMP-V-3 by further separation, screening, and structure identification. Among these 12 peptide sequences, 4 peptide sequences ERFNVE, TELVLK, MELVLK, and FDDKLD have lower binding energy and more interaction binding bonds force to 1O8A. The peptides with hydrophobic amino acids account for a large proportion and contribute more to its ACE inhibitory. This is the first report on four novel ACE inhibitor peptides, produced from the hydrolysates of HMP using Flavourzyme, which have potential applications in several industries.

## Data availability statement

The original contributions presented in this study are included in the article/supplementary material, further inquiries can be directed to the corresponding authors.

## Author contributions

YC, YZ, and WW conceived and advised on all aspects of the study. YC, YZ, QQ, FL, and XL performed experiments, analyzed data, and wrote the manuscript. YJ and WW supervised all aspects of the study. QC, NW, KB, and SS completed language changes. XW put forward the advice. All authors discussed and commented on the manuscript.
